# The preterm gut microbiota and administration routes of different probiotics: a randomized controlled trial

**DOI:** 10.1038/s41390-023-02560-y

**Published:** 2023-04-05

**Authors:** Ella-Noora Rahkola, Samuli Rautava, Henni Hiltunen, Chandler Ross, Leo Lahti, Erika Isolauri

**Affiliations:** 1 Department of Clinical Sciences, Faculty of Medicine, University of Turku, Turku, Finland; 2 Department of Paediatrics and Adolescent Medicine, Turku University Hospital, Turku, Finland; 3 Department of Pediatrics, University of Helsinki and Children’s Hospital, Helsinki University Hospital, Helsinki, Finland; 4 Department of Computing, University of Turku, Turku, Finland

## Abstract

**Background:**

Preterm children with their aberrant gut microbiota and susceptibility to infections and inflammation constitute a considerable target group for probiotic therapy to generate the age-appropriate healthy microbiota.

**Methods:**

68 preterm neonates were randomized into five intervention groups: Beginning from the median age of 3 days, 13 children received *Lactobacillus rhamnosus* GG (LGG) directly orally, and 17 via the lactating mother. 14 children received LGG with *Bifidobacterium lactis* Bb-12 (Bb12) orally, and 10 via the lactating mother. 14 children received placebo. The children’s faecal microbiota was assessed at the age of 7 days by 16S rRNA gene sequencing.

**Results:**

The gut microbiota compositions of the children directly receiving the probiotic combination (LGG + Bb12) were significantly different from those of the children receiving the other intervention modes or placebo (*p* = 0.0012; PERMANOVA), the distinction being due to an increase in the relative abundance of *Bifidobacterium animalis* (*P* < 0.00010; ANCOM-BC), and the order *Lactobacillales* (*P* = 0.020; ANCOM-BC).

**Conclusion:**

The connection between aberrant primary gut microbiota and a heightened risk of infectious and non-communicable diseases invites effective microbiota modulation. We show that the direct, early, and brief probiotic intervention of LGG + Bb12 10^9^ CFU each, is sufficient to modulate the gut microbiota of the preterm neonate.

**Impact:**

- Preterm children have a higher risk of several health problems partly due to their aberrant gut microbiota.
- More research is needed to find a safe probiotic intervention to modify the gut microbiota of preterm children. The maternal administration route via breast milk might be safer for the newborn.
- In our study, the early and direct administration of the probiotic combination Lactobacillus rhamnosus GG with Bifidobacterium lactis Bb-12 increased the proportion of bifidobacteria in the preterm children’s gut at the age of 7 days, but the maternal administration route was not as effective.

## Introduction

The gut microbiota of healthy term vaginally delivered breast-fed children is rich in bifidobacteria.^1^ Preterm children, in turn, have aberrant gut microbiota which accommodate high abundances of facultative anaerobic bacteria, especially *Enterobacteriaceae* and *Enterococcaceae.*^2^ Additionally, as compared to full-term neonates, the preterm neonatal microbiota exhibit lower biodiversity, a greater abundance of possibly pathogenic bacteria, and a smaller abundance of anaerobic bacteria.^2–4^

The establishment of the gut microbiota guides the maturation of key regulatory systems during the critical period of developmental plasticity.^5^ In this process, the neonate acquires a dual function, namely, a delicate balance of effective alertness to pathogens while concomitantly maintaining disease-free coexistence with the indigenous microbiota, endorsing the anti-inflammatory tone of the gut barrier. The equilibrium is a result of co-evolution between several species.^6^ The composition and functions of aberrant primary microbiota hamper this interaction, generating sustained local inflammation and gut barrier dysfunction, which in turn leads to a heightened risk of acute infections and non-communicable diseases such as asthma and obesity as long-term sequelae.^7,8^ Hence, the microbiota of healthy term children may be taken as a model to increase preterm children’s resilience to the myriad of detrimental environmental exposures the preterm child faces during the critical period of maturation.^8^

The use of specific probiotics has received considerable attention, particularly in the attempt to fight the acute sequelae of prematurity. A Cochrane meta-analysis with more than 10,000 preterm neonates determined that administering specific probiotics may prevent necrotizing enterocolitis, although they expressed the need for more studies to be carried out.^9^ Additionally, the European Society for Paediatric Gastroenterology Hepatology and Nutrition has made a concordant conditional recommendation.^10^

Probiotics, by definition, are live microorganisms^11^ and therefore the major concern is related to their administration to immature and immunologically inexperienced preterm children.^9^ Some cases of bacteremia associated with specific probiotics have been reported^12^ as well as gastrointestinal mucormycosis from *Rhizopus oryzae ‐*contaminated probiotic.^13^ This risk may be circumvented by administering the probiotic to the mother instead of the child, profiting from the vertical transmission of microbial elements^14,15^ and the promotion of anti-inflammatory features of breast milk.^16–19^

The data on how the microbiota of a preterm child responds to probiotic intervention, directly administered to the child as compared to the administration via mother, are lacking. Consequently, we compare these modes of intervention, and secondly, seek probiotic strain combinations that give the optimal microbiota modulation for this vulnerable population.

## Methods

**Fig. 1:**
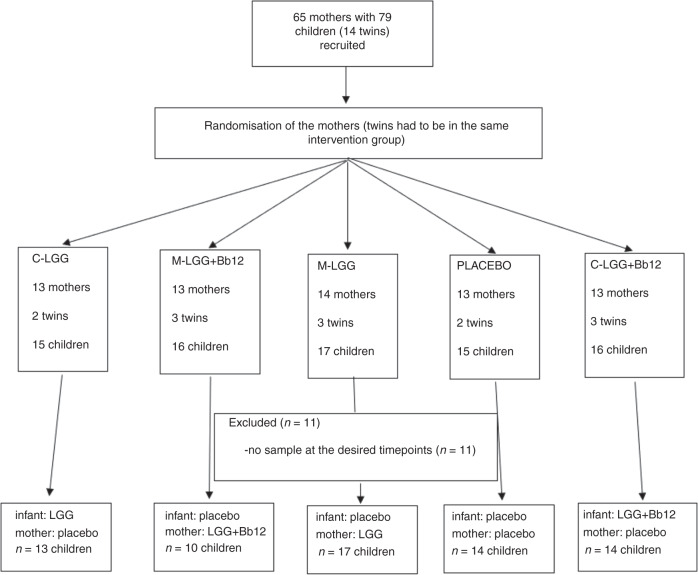
The progress of the study. Initially, 65 mothers with their 79 newborn children were randomized into five intervention groups. Eventually, 68 mother-child pairs were eligible for the study. The abbreviations of the intervention groups are described in figure.

A randomized, double-blind, placebo-controlled clinical trial recruited preterm neonates and their mothers at the Turku University Central Hospital from April 2014 to March 2018. The trial was registered in October 2011 (ClinicalTrials.gov Identifier NCT01454661). Children born during a 25–35-week gestational period, younger than 3 days of age, admitted in the Neonatal Intensive Care Unit (NICU), and those who were able to provide a faecal sample at the age of 7 days were eligible for the study. The exclusion criteria were severe birth asphyxia and significant anomalies in the gastrointestinal tract. In total, 68 mother-child pairs were accepted into this study (Fig. 1). The number of subjects was determined by practical factors, including cost and feasibility of sample analyses. The Ethics Committee of the Hospital District of Southwest Finland found the study acceptable.

The intervention strains were *Lactobacillus rhamnosus* GG (ATCC 53103, currently defined as *Lacticaseibacillus rhamnosus* GG, abbreviation LGG) or LGG in combination with *Bifidobacterium lactis* Bb-12 (Bb12). The placebo product comprised of microcrystalline cellulose. The supplements were produced and kindly provided without cost by Dicofarm S.p.A. The mothers with their children were randomized into one of the five intervention groups (Fig. 1):

-Placebo: both the mother and the child received placebo

-C-LGG: The mothers received placebo, the children were administered LGG.

-C-LGG + Bb12: mother: placebo, child: LGG + Bb12

-M-LGG: mother: LGG, child: placebo

-M-LGG + Bb12: mother: LGG + Bb12, child: placebo

Random permuted block randomisation was performed by an independent statistician and the randomisation code was generated by SAS, version 9.3 for Windows. The allocation concealment was ensured using sequentially numbered containers. The University laboratory and the Hospital pharmacy held the emergency envelope to be opened in case of serious adverse event or disease in a study subject. Clinical research nurses recruited the patients and gave the intervention products in numerical order.

The children received the intervention substances as probiotic capsules the contents of which were dispersed with a small amount of breastmilk and administered with their morning feed throughout their stay in the NICU. The mothers received the probiotics as capsules. The daily doses of the probiotics were 10^9^ CFU each. The age of the children, when the administration of the intervention product was begun, is described in Table 1. In summary, 35 children (51%) started to receive the supplement at the age of 3 days, 45 children (69%) at the age of 3 days or younger, and 59 (87%) at the age of 4 days or younger. Three children in the group C -LGG + Bb12 did not receive the study supplement before the faecal sample was taken on the 7th day. Their mothers started to receive the placebo product when the children were 3 days old.

**Table 1:** The age of the children (days) when the administration of the intervention product was begun (median and range) by intervention group.

Intervention group	Starting age (median)	Starting age (range)
M-LGG	3	1–6
M-LGG + Bb12	3	2–5
C-LGG	3	2–4
C-LGG + Bb12	4	2–8
Placebo	3	2–4

The gut microbiota compositions of the study subjects (68 children) were assessed using faecal samples obtained 7 days after birth. The samples were stored in RNAlater solution at −80 °C. The gut microbiota compositions were assessed by analyzing the DNA isolated from faecal samples and utilizing 16S rRNA sequencing. The DNA was extracted by homogenizing 100–125 mg of faeces in the lysis buffer via bead beating with FastPrep-24 (MP Biomedicals, Irvine, CA). Then, commercial kit InviMag Stool DNA Kit (Stratec Molecular, Berlin, Germany) was used with the automated KingFisher DNA System (Thermo Fisher Scientific Oy, Vantaa, Finland). The manufacturer’s protocol was followed, and it included nucleic acid binding on magnetic beads, five-step washing, and elution. A Qubit® 2.0 Fluorometer (Life Technology, Carlsbad, CA) was used to measure the total DNA consentration. V3–V4 region of the 16S rRNA gene was amplified following the 16S rDNA gene Metagenomic Sequencing Library Preparation Illumina protocol (Cod. 15044223 Rev. A). The multiplexing was completed by using Nextera XT Index Kit (FC-131–2001). One microliter of the PCR product was run on a Bioanalyzer DNA 1000 chip to verify the size; the expected size being ca. 550 bp on a Bioanalyzer trace. The sequencing was made by utilising a 2 × 300 bp paired-end run (MiSeq Reagent Kit v3) on a MiSeq-Illumina platform following the manufacturer’s protocol. Both positive and negative sequencing controls were used to exclude contamination and batch effects. These sequencing results are available from the corresponding author as Fastq files upon reasonable request. In 16S rRNA sequencing method, the 16S rRNA gene, which codes the small subunit of procaryotic ribosome, is copied. Subsequently, these copies are grouped into coherent Amplicon Sequence Variants (ASVs) and mapped to a reference database for downstream compositional data analysis at the higher taxonomic levels. In our study, the QIIME2 (version 2019-07 and 2020-11) pipeline was used to analyze the raw sequences. Phred33-importing tool for paired-end data was used to import the data and DADA2 software package to correct Illumina-sequenced amplicon errors.^20^ The Greengenes v.13.8 database with 99% ASV taxonomic classifier was used to create a phylogenetic tree. The faecal samples were processed at the Functional Foods Forum of the University of Turku and sequenced at FISABIO-GVA through the Institute of Agrochemistry and Food Technology in Valencia, Spain.

CONSORT 2010 Checklist is included in the supplementary files (Supplementary File 1).

### Statistics

The data was stored as a TreeSummarizedExperiment^21^ (TSE) object (TreeSummarizedExperiment 2.0.3), in R (BiocManager—1.30.16, BiocVersion—3.13.1). The analysis thereon was carried out using the R/Bioconductor packages ‘mia’^22^ (1.1.14), and ‘phyloseq’^23^(1.36.0). We investigated the distribution of samples via an MDS plot at the species level using the Aitchison distance (CLR-transformed abundances + Euclidean distance). We used Permutational Multivariate Analysis of Variance (PERMANOVA) to assess the significance of the differences present at the species-level community composition using the ‘adonis’ function from the ‘vegan’ R package (vegan – 2.5-7). We then used ANCOM-BC^24^ (ANCOM-BC 1.2.2) to identify differentially abundant taxa between the groups, using the Bonferroni method to correct for multiple testing.^25,26^ The groups were further analyzed in a post-hoc comparison using the ‘calc pairwise permanovas’ function from the ‘mctoolsr’ R package (mctoolsr − 0.1.1.2). We also performed the PERMANOVA analysis on other factors in the metadata including gestational age, mode of delivery, and sex of the child to assess their potential significant association with the outcomes.

## Results

**Fig. 2:**
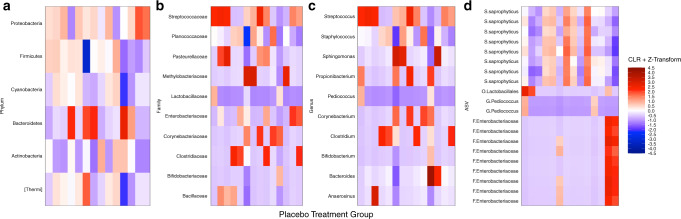
A heatmap of the gut microbiota of the preterm children receiving placebo. The counts of the bacterial groups have been converted into relative abundances, then CLR-transformed for the samples, and Z-transformed for the features (taxa) so that the heatmap is distributed around 0. The *y*-axis shows every phylum (**a**), the 10 most common families in these samples (**b**), the 10 most common genera in these samples (**c**), and the 20 most common ASVs in these samples (**d**) associated with the most specific accuracy achievable by using the Greengenes database. The accuracy which was possible to achieve is described by letters G for genus, F for family and O for order.

The clinical characteristics of the study population are summarized in Table 2. A heatmap of the gut microbiota of the preterm children receiving placebo is described in Fig. 2. The unmodulated gut microbiota of the preterm children are rich in *Staphylococcus saprophyticus* while there are few bifidobacteria. We documented that sex, gestational age, and birth mode did not have a significant effect on the gut microbiota in this population (Table 3).

**Table 2:** Characteristics of the children at birth by intervention group. Median with range or number with percentage.

	Placebo	M-LGG	M-LGG + Bb12	C-LGG	C-LGG + Bb12	All children
Gestational age (weeks)	33.0 (27.7–34.4)	32.7 (27.7–34.3)	32.4 (27.6–34.6)	32.1 (28.9–34.4)	32.2 (25.7–34.7)	32.0 (25.7–34.7)
Birth weight (g)	2185 (1320–3030)	1570 (755–2670)	1793 (680–2760)	1600 (1250–2426)	1560 (460–3050)	1783 (460–3050)
Birth mode						
Vaginal	14 (100%)	11 (65%)	4 (40%)	4 (31%)	6 (43%)	39 (57%)
Section	0 (0%)	6 (35%)	6 (60%)	9 (69%)	8 (57%)	29 (43%)
Sex of the child						
Girl	7 (50%)	11 (65%)	5 (50%)	9 (69%)	7 (50%)	29 (43%)
Boy	7 (50%)	6 (35%)	5 (50%)	4 (31%)	7 (50%)	39 (57%)

**Table 3:** The effect of possible confounding factors on the gut microbiota.

Variable/Taxonomical level	Phylum	Family	Genus	Species	ASV
Gestational age	0.036 (0.11)	0.031 (0.077)	0.031 (0.080)	0.030 (0.083)	0.020 (0.083)
Delivery mode	0.024 (0.20)	0.042 (0.20)	0.041 (0.20)	0.040 (0.20)	0.045 (0.20)
Sex of the child	0.045 (0.20)	0.018 (0.20)	0.018 (0.20)	0.022 (0.20)	0.020 (0.20)

The R2 values that describe to what extent the gestational age, delivery mode, and sex of the child explain the variance in different bacterial taxonomic levels in the gut microbiota. In brackets, the *p*-values of the comparisons between the intervention groups made according to the study subjects’ features concerning possible confounding factors using PERMANOVA. Gestational age, delivery mode (vaginal or caesarean section), and sex of the child were not associated with the gut microbiota composition.

All children received their own mothers’ breastmilk. On average, they started to receive it at the age of 1.3 days. All but one child (in group M -LGG + Bb12) received additionally donor breast milk, commonly starting on the day they were born, except for one child who was given donor milk at the age of 3 days (in group C -LGG + Bb12). Five children received formula at discharge, but this happened after the faecal samples were obtained at the age of 7 days. These were subjects from groups C -LGG, Placebo (3 children), and C -LGG + Bb12. According to the hospital guideline, all children with birth weight below 1800 g received breast milk fortifier until their weight was ca. 3500 g.

The children and their mothers received antibiotics if needed according to the hospital’s protocol, which are well in line with the current clinical practice. Consequently, only 3 of 68 children (in groups C -LGG, M -LGG, and Placebo) did not receive any antibiotics during the study. The children usually received benzylpenicillin with gentamicin. The mothers of 43 children received intrapartum antibiotics, the most common reason being prophylaxis (usually benzylpenicillin or cefuroxime with or without azithromycin) due to caesarean section. The number of the children whose mothers received intrapartum antibiotics in each group were: C -LGG: 7/13 children, M -LGG + Bb12: 8/10 children, M -LGG: 10/17 children, Placebo: 8/14 children, C -LGG + Bb12: 10/14 children.

**Fig. 3:**
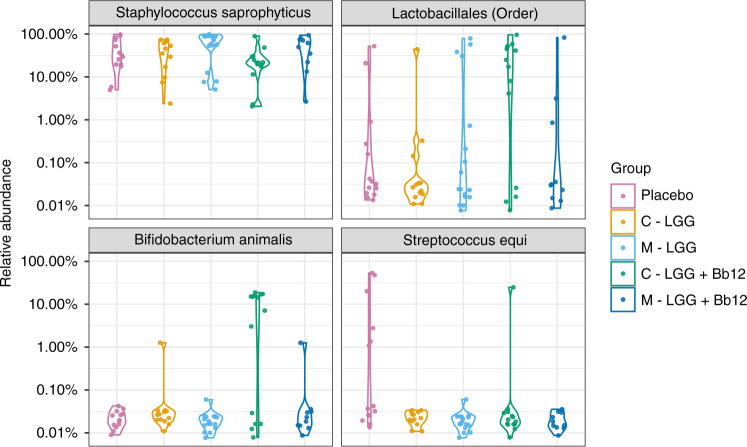
The microbiota of the children: the relative abundances (%) of 4 ASVs named by the bacterial groups they correspond described as violin plots. The ASVs of which relative abundances were statistically different between intervention arms are described here. The mean relative abundances and the p-values of the comparisons between the intervention groups are listed in Table 4.

The choice of the probiotic strain and the route of administration had a definite effect on the children’s gut microbiota. The gut microbiota compositions of the children directly receiving the probiotic combination (LGG + Bb12), in particular, were significantly different from those of the children receiving the other intervention modes or placebo (*p* = 0.0012; PERMANOVA) (Fig. 3 and Supplementary File 2). This distinction was due to an increase in the relative abundance of *Bifidobacterium animalis* and the ASV associated with the order *Lactobacillales* (*P* = 0.020; ANCOM-BC) (Table 4). While this ASV was the most abundant one in the gut microbiota of the children receiving directly both LGG and Bb12, neither the family, genus, nor species of *Lactobacillales* could be determined more precisely by using the Greengenes reference database. In the other intervention groups, the most abundant bacterial species was *Staphylococcus saprophyticus*.

**Table 4:** The mean relative abundances (%) of different bacterial groups connected to specific ASVs in the gut microbiota and the *p*-values of the comparisons to the double placebo group (ANCOM-BC).

Group		Placebo	C-LGG + Bb12	C-LGG	M-LGG + Bb12	M-LGG
*Bifidobacterium animalis*						
	Relative abundance (%)	0	9.0^a^	0.1^b^	0.1	0^b^
	*p*-value, difference to the placebo group	–	<0.00010	1.0	1.0	1.0
*Staphylococcus saprophyticus*						
	Relative abundance (%)	43.0	26.2^c^	43.1	51.4	62.2^c^
	*p*-value, difference to the placebo group	–	1.0	1.0	1.0	1.0
*Lactobacillales* (order)						
	Relative abundance (%)	5.4	29.0^d^	3.6^d^	8.8	12.4
	*p*-value, difference to the placebo group	–	0.020	1.0	1.0	1.0
*Streptococcus equi*						
	Relative abundance (%)	9.2	1.8	0	0	0
	*p*-value, difference to the placebo group	–	0.44	0.015	0.015	0.012

The ASVs of which relative abundances were statistically different between intervention arms are described here described by the names of the bacterial groups they correspond.

^a^There were also statistically significantly more *Bifidobacterium animalis* in the group C-LGG + Bb12 than in the groups C-LGG (*p* < 0.0001), M-LGG + Bb12 (*p* = 0.0079), and M-LGG (*p* < 0.0001).

^b^There were statistically significantly more *Bifidobacteria animalis* in the group C-LGG than in the group M-LGG (*p* = 0.040).

^c^There were statistically significantly more *Staphylococcus saprophyticus* in the group M-LGG than in the group C-LGG + Bb12 (*p* = 0.0072).

^d^There were statistically significantly more ASVs associated with the order Lactobacillales in the group C-LGG + Bb12 than in the group C-LGG (*p* = 0.00050).

Maternal administration of the probiotic combination LGG + Bb12 was not as effective as the direct route to increase the amount of *Bifidobacterium animalis* in the children’s gut microbiota (*P* = 0.0079; ANCOM-BC), although it produced detectable levels of *Bifidobacterium animalis*. It also failed to increase the number of *Lactobacillales* in the gut.

The direct administration of LGG alone increased the number of *Bifidobacterium animalis* to a greater extent than the maternal administration of the strain (*P* = 0.040; ANCOM-BC) (Table 4), albeit by a smaller amount compared to the directly administered combination of LGG + Bb12 (*P* < 0.00010; ANCOM-BC). Moreover, no augmentation of the abundance of *Lactobacillales* was achieved by the direct administration of LGG alone.

## Discussion

In early life, the profile of the gut microbiota of a full-term, vaginally delivered, breast-fed infant is considered the gold standard.^27^ The genus *Bifidobacterium* comprises the predominant group of the microbiome of healthy term infants,^28^ who also remain healthy long-term.^29,30^ We describe here a restoration of the gut microbiota in the preterm neonate towards a composition rich in bifidobacteria, which have been proved effective in reducing the intestinal inflammatory state by regulating pro- and anti-inflammatory cytokines.^31,32^ The present results corroborate those of previous studies documenting that specific probiotics may transiently colonize the infant gut, modulate the preterm child´s gut microbiota composition,^33^ and extend these to the documentation of the optimal mode of administration.

Our study has a number of limitations. The intervention groups were small because several different interventions were included. Nevertheless, our study is of importance for future research on the administration routes of probiotics as no similar studies have been carried out before. The children started to receive the supplementation at the median age of 3 days and the faecal samples were collected at the age of 7 days. The short time interval between the commencement of the intervention and sample collection may not reveal the full extent of the effect of the probiotic supplementation. Albeit, the short and early intervention was sufficient to modulate the gut microbiota of the preterm infant when administered directly but not through the mother. The adequate dosage of probiotics is not yet known.^34^ We showed that the dosage of 10^9^ CFU of each probiotic was sufficient to modulate the preterm neonate’s gut microbiota when administered directly, but not through the lactating mother. It is not excluded that a higher dosage would be more efficient. Consequently, more studies are needed to determine this. The bacterial composition of faecal samples is widely used to represent the gut microbiota as this method is non-invasive. It has been proven that there is a strong positive correlation between the presence of bifidobacteria in the colon and in the faecal samples.^35^ However, the microbiota of faecal samples may be biased toward colonic bacteria, such as bifidobacteria compared to *Lactobacillus* species which usually live in the small intestine.^36^ Furthermore, the children’s faecal samples were collected from their diapers, hence contamination from skin bacteria can not be excluded. A child’s cutaneous microbiota is rich in *Staphylococcus, Streptococcus, Corynebacterium* and *Prevotella.*^37^ Some studies prove that the amount of viable bacteria in probiotic supplementations reported by the manufacturer sometimes differs from the amount measured by the researchers.^38^ We did not verify the amount of bacteria in the probiotic products by qPCR.

The results of this study bespeak differences in early gut microbiota in preterm and full-term children. Our results, in agreement with those of previous studies,^2–4^ establish that the unmodulated microbiota of the preterm children is typified by facultative anaerobic and possibly pathogenic species such as *Staphylococcus saprophyticus* while bifidobacteria tend to be absent. This shift in the gut microbiota composition may arise from the prematurity per se, or alternatively, be connected with the early hospital environment exposures, reduced skin contact, later onset of breastfeeding, and a higher likelihood of delivery by caesarean section and of antibiotic contacts.^3^ Aberrant primary gut microbiota composition hampers the step-wise compositional development of the gut microbiota as bacterial species modify their shared habitat.^1,39^ Moreover, dysbiosis during the critical period of maturation may lead to lasting alterations in the immune and metabolic phenotype of the child thereby furnishing one explanation for the heightened risk of chronic inflammatory non-communicable diseases.^40^ In fact, in addition to neonatal morbidity and mortality, preterm birth has been associated with significant long-term sequelae including impaired growth, neurodevelopmental, pulmonary, and gastrointestinal problems as well as increased risk of cardiovascular and metabolic diseases.^41^

Taken previous studies - mostly in full-term children – into account, it appears that specifically a greater number of bifidobacteria among the early gut microbiota is associated with a lower risk and shorter duration of acute infections,^42^ and a reduced risk of obesity, autoimmune diseases, atopic eczema and wheeze, or celiac disease later in life.^1,29^ Bifidobacteria have been proved effective in reducing the intestinal inflammatory state by regulating pro- and anti-inflammatory cytokines.^31,32^ Additionally, members of the genus *Bifidobacterium* are thought to be keystone species in the neonatal gut as they are able to utilize human milk oligosaccharides producing and maintaining an anaerobic and acidic gut environment for other bacterial species, thus preventing enteropathogenic infection and an inflammatory intestinal state.^39,43^ Conversely, immature gut microbiota composition has been associated with restricted growth in malnourished children despite adequate energy restoration.^44^ This same phenomenon may hold true in the preterm child; faecal microbiota transplants of meconium from preterm children transplanted to germ-free mice were shown to reproduce the phenotype of restricted growth in the recipient.^45^ The gut microbiota contributes to the mechanisms of the sustained inflammation causing gut barrier dysfunction^46^ thereby evoking systemic inflammation^47^ and enabling the suppression of growth hormones.^48^ But then again, dysbiosis and low-grade systemic inflammation represent also a central prerequisite for a cluster of over-weight-associated pathologies.^49^ Thus, the shift in the primary gut microbiota composition may represent an early indicator of growth impairment and early excessive weight gain, the two sides of malnutrition.^50,51^

Specific probiotic bacteria have been shown to stabilize the gut microbial environment and the permeability barrier of the intestine, and to enhance systemic and mucosal IgA responses.^52^ These are important effects in relation with preterm children who are particularly susceptible to infections and inflammation as the immature enterocytes preferably respond to intraluminal antigens with proinflammatory cytokines.^53^ Specific probiotics have been shown to transiently colonize 86% of the preterm infant gut at the age of 7 days if the direct probiotic supplementation began from one to 3 days after birth.^54^ Similarly, in our study, the probiotic combination of LGG + Bb12 increased the proportion of bifidobacteria in the gut at the age of 7 days. Another previous study^55^ documented that a supplement containing several species of lactobacilli and bifidobacteria plus fructo-oligosaccharides administered for 4 weeks beginning at the age of younger than 7 days led to a bifidobacterial-rich microbiota in 64% of preterm infants. During the first week of the intervention, only 1 child out of 11 was colonized. This lower proportion of successful colonization might be due to heterogenicity in the initiation of the intervention as some of the study subjects apparently began to receive the supplement when they were closer to the age of 7 days. As a result, timing may be of essence to abate the effects of detrimental exposures in the early extrauterine environment of the preterm child. The child’s aberrant gut microbiota should be treated early as it may be one of the crucial factors participating in the child’s immunologic and metabolic programming affecting their later health.^56^ The child’s gut seems to be unstable and thus modifiable early in life as no differences were observed in the gut microbiota during the first week of life between vaginally delivered children and children born via caesarean section but after 2 weeks certain differences were noticeable.^57^ Likewise, a previous study^58^ assessed the longitudinal changes of the populations of bifidobacteria in preterm children’s guts during their first year of life and concluded that there are fluctuations in the species, strains, and the gene expression of bifidobacteria, indicating that early external factors, or probiotic interventions, are able to modify the preterm microbiota. Indeed, it is not too late to begin the modification of the preterm child’s microbiota after birth. When begun during pregnancy and continued during breast-feeding, maternal administration of probiotics has been proven effective and safe in reducing the risk of atopic eczema and sensitization promoting the immunoprotective potential of breast milk in children born at full term.^59,60^ In our study, the maternal administration of probiotics did not modulate the microbiota towards a composition rich in bifidobacteria in preterm children, but the administration began only after delivery. In agreement with an earlier study in preterm children,^55^ lactobacilli without bifidobacteria did not increase the proportion of bifidobacteria in the children’s guts, even though previous results in infants document such bifidogenic effects.^61^ The colonization of the children’s gut by LGG has been observed in earlier studies^62^ even when administered via the lactating mother.^63^ In our study, LGG was not detected in any of the intervention groups but it may be possible that it was one of the unknown species of the order *Lactobacillales* abundant in the gut microbiota of the children directly receiving both LGG and Bb12. In that event, Bb12 would have been required to increase the number of LGG.

No adverse effects were observed in our study even if preterm neonates were directly administered living bacteria. This observation is strengthened by previous findings of the safe prophylactic use of LGG in preterm infants over a 12-year period.^64^ By administering preterm children directly the probiotic combination (containing *Lactobacillus* and *Bifidobacterium* strains, previously documented safe and effective) once a day, beginning from the first enteral feed, it is possible to increase the number of bifidobacteria in these children’s gut microbiota by 7 days of age.

### Supplementary Information

Checklist item

SUPPLEMENTARY FILE

## Data Availability

The datasets generated and analysed during the current study are available from the corresponding author on reasonable request. Source code for the analyses is available online [10.5281/zenodo.6674816].
