# Supplementary material for: The preterm gut microbiota and administration routes of different probiotics: a randomized controlled trial
Source: Pediatr Res. 2023 Apr 5;94(4):1480–7. doi: 10.1038/s41390-023-02560-y (PMC10589095; doi:10.1038/s41390-023-02560-y)
Supplement: Supplementary file 2 — SUPPLEMENTARY FILE [file 41390_2023_2560_MOESM2_ESM.pdf]

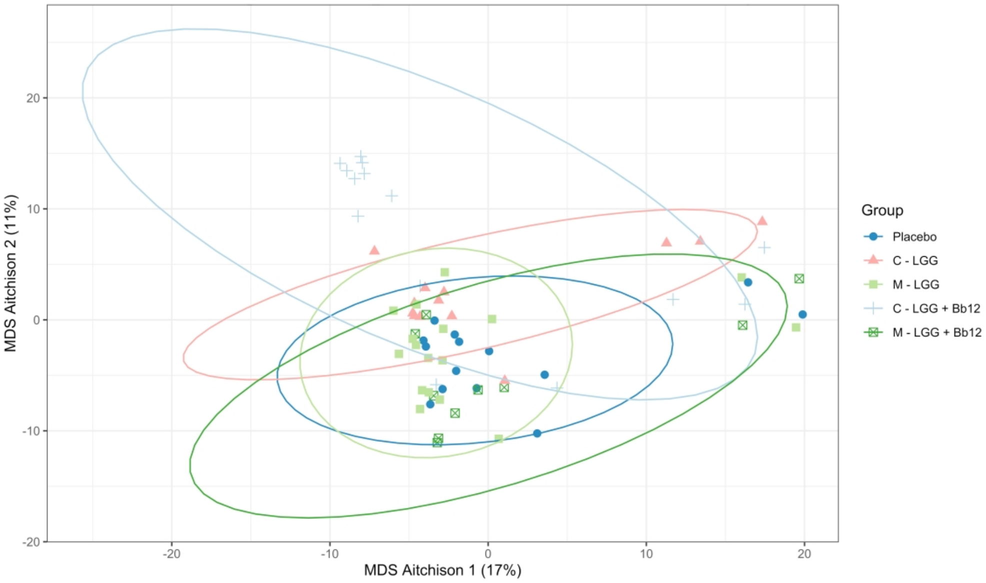

SUPPLEMENTAL FILE 2 The similarity of the gut microbiota of the study subjects. The members of the same intervention group share the same symbol. The closer the symbols are to each other, the more similar the compositions of the gut microbiota are. The children who directly received the probiotic combination Bb12 + LGG tended to cluster.

190x112mm (600 x 600 DPI)
